# Design of broadband graphene-metamaterial absorbers for permittivity sensing at mid-infrared regions

**DOI:** 10.1038/s41598-018-22536-x

**Published:** 2018-03-08

**Authors:** Hailong Huang, Hui Xia, Wenke Xie, Zhibo Guo, Hongjian Li, Ding Xie

**Affiliations:** College of Physics and Electronics, Central South University, Changsha, 410083 China

## Abstract

In this paper, a tunable broadband metamaterial absorber (MA) based on graphene is investigated theoretically and numerically at mid-infrared regions. Compared with the previously reported multiband graphene-based MAs, a broad bandwidth of 11.7 THz with the absorption over 90% is obtained in the proposed MA, which is composed of a Jerusalem cross (JC) metal encrusting into the slot graphene layer in the top layer. The results show that the origin of broadband absorption is caused by coupling effect between metal and graphene, and this effect is explained by the two-mode waveguide coupling theory. The tunability of MA is achieved via changing the external gate voltage to modify the Fermi energy of graphene. Further results show that the proposed MA can be used as the permittivity sensor with a high absorption. This work indicates that the proposed MA has the potential applications with respect to sensors and infrared absorbers.

## Introduction

Metamaterial absorbers (MAs), a new type of artificial materials, have been widely investigated in recent years due to the electromagnetic (EM) properties which are absent in traditional materials^1,2^. The properties are used to realize some new physical functions like negative refraction index^3^, perfect lensing^4^, bolometers^5^, and PIT effect^6–11^, which are caused by sub-wavelength metallic resonators. Landy *et al*.^12^ demonstrated that the overall dimensions of MA can be designed to offer perfect absorption at microwave frequencies owing to the matched impedance to free space. Since then, lots of devices^13–16^ have been proposed and studied from the microwave^17^ to terahertz^18^ regions in order to obtain perfect absorption^19^, polarization-insensitive^20^, wide angle^21^ and tunability^22^. The single-band high absorption is unsuitable in practical applications^23^. Hence different methods have been proposed to induce a multi-band absorption, such as loading lumped elements^24^, introducing the asymmetric structure^25^, and using two circular rings with different radius^26^. Recently, using hybrid materials in the different layers is another way to realize the broadband or multiband absorption at THz regions. For example, Ling *et al*.^27^ designed a broadband polarization-insensitive MA based on hybrid materials (SiO_2_ and metal) in the middle layer, which induces a high absorption from 60.5 to 115.5 THz. Zhou *et al*.^28^ reported a two-dimensional (2D) absorber using Si_3_N_4_ and Al, and exhibiting a perfect absorption over the whole visible region. However, these structures are suffered from some challenges as follows: (i) the tunability is obtained only by reconstructing the geometric structure, which is inappropriate for making the tunable devices^29,30^. (ii) The increased thickness is occurred when the hybrid materials are used to make broadband MA. Therefore a more straightforward and convenient strategy should be developed to guide the design of tunable MAs with a thinner thickness.

Graphene has attracted worldwide interests as a promising platform for building perfect tunable MAs due to its unique properties as follows^31^: (i) its electric conductivity can be continuously tuned in a broad frequency range, (ii) the electrical tunability of graphene enables fast reflection or absorption modulation, (iii) graphene is a two-dimensional material, which makes it more suitable for planar structure, (iiii) graphene consists of one monolayer of carbon atoms, which means the thickness of the MA composed of graphene is thinner than that composed of the metal. Recently, lots of multi-band graphene-based MAs have been proposed^32–35^. However, a broadband high absorption is not obtained in the graphene-based devices.

In this paper, a tunable graphene-based MA is proposed numerically and theoretically to realize the broadband absorption at mid-infrared regions. This absorber consists of three layers: a Jerusalem cross (JC) metal, which is embedded into the slot graphene plane at the top layer, a dielectric spacer, and the bottom gold film. The results show that a broad bandwidth of 11.7 THz with absorption over 90% is obtained due to the coupling effect between metal and graphene, and the origin of this effect is explained by using the two-mode waveguide coupling theory. The graphene’s Fermi energy can be dynamically tuned via changing the external gate voltage to realize the tunability of MA. Furthermore the proposed system has the advantage of high absorption for permittivity sensing application. Therefore this MA may find its potential applications in sensors and infrared absorbers.

## Structure and Simulation

**Figure 1:**
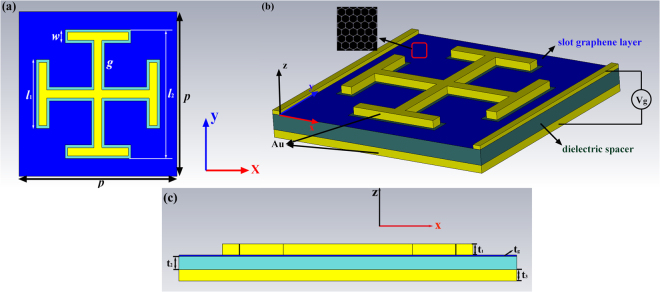
The unit cell of the proposed MA: (**a**) front view (**b**) perspective view (**c**) bottom view;

The unit cell of the MA is shown in Fig. 1. The first layer is composed of a square graphene film array and then patterned into the JC slot by photo-etching. Subsequently a JC metal is employed to be embedded into this slot. This layer is used to induce the broadband high absorption. The second layer is a slab made of dielectric spacer, which has a relative permittivity of 3.5 and a loss tangent of 0.057^36^. The third layer is metal plate, which is placed at the backside of dielectric layer and acting as an electrode for biasing the graphene layer as shown in Fig. 1(b). The metal is set as gold with the electric conductivity of *σ* = 4.09 × 10^7^ S/m. The optimized parameters of the unit cell are listed as follows: *p* = 3 μm, *w* = 0.25 μm, *g* = 0.05 μm, *l*_1_ = 1.25 μm, *l*_2_ = 2.35 μm, *t*_1_ = *t*_2_ = *t*_3_ = 0.1 μm. Our results are obtained through a full wave EM simulation based on finite integration technique (FIT) and finite element method (FEM) owing to their accuracy in results and its suitability for the study of high frequency and three-dimensional EM structures^37–39^. In the *x*- and *y*- directions, the unit cell boundary conditions are utilized, and open space boundary conditions are applied in the *z*-direction. When the incident wave is vertical to the upper surface of the MA, the absorption *A* is obtained from the S-parameters by *A* = 1 − |*S*_21_|^2^ − |*S*_11_|^2^, where *S*_11_ is the reflection coefficient and *S*_21_ is the transmission coefficient. Due to the thickness of the gold is much larger than its skin depth, the transmission coefficient *S*_21_ = 0. Therefore the expression can be simplified as *A* = 1 − |*S*_11_|2.

The complex surface conductivity of graphene (0.5 nm in thickness) can be calculated from the Kubo formula and is described with interband and intraband contributions as^40^:

1 $${\rm{\sigma }}(\omega ,{E}_{{\rm{f}}},{\rm{\Gamma }},{\rm{T}})={\sigma }_{{\rm{inter}}}+{\sigma }_{{\rm{intra}}}$$

2 $${\sigma }_{{\rm{intra}}}=\frac{2{k}_{b}T{e}^{2}}{\pi {{\rm{\hbar }}}^{2}}\,\mathrm{ln}(2\,\cosh \,\frac{{E}_{{\rm{f}}}}{2{k}_{b}T})\frac{i}{(\omega +i\Gamma )}=\frac{\alpha }{-i\omega +\Gamma }$$

3 $${\sigma }_{{\rm{inter}}}=\frac{{e}^{2}}{4{\rm{\hbar }}}[H(\frac{\omega }{2})+i\frac{4\omega }{\pi }{\int }_{0}^{\infty }\frac{H({\rm{\Omega }})-H(\frac{\omega }{2})}{{\omega }^{2}-4{{\rm{\Omega }}}^{2}}d{\rm{\Omega }}]$$

where

$${\rm{H}}({\rm{\Omega }})=\,\sinh (\frac{{\rm{\hbar }}{\rm{\Omega }}}{{k}_{b}T})/[\cosh (\frac{{\rm{\hbar }}{\rm{\Omega }}}{{k}_{b}T})+\,\cosh (\frac{{E}_{{\rm{f}}}}{{k}_{b}T})]$$

*ω* is the angular frequency, *E*_f_ is the Fermi energy of graphene, *Γ* is the collision angular frequency, *T* = 300 K is the temperature, *K*_b_ is the Boltzmann constant, *e* is the elementary charge, and ħ is the reduced Planck’s constant. When the Fermi level is larger than half of the photon energy, the intraband contributions dominate the graphene conductivity as the interband transitions are negligible due to Pauli blocking^41^. In this work, *E*_f_ is controlled by external gate voltage (*V*_g_), and shown as following equation:

4 $${E}_{f}={\rm{\hbar }}{v}_{f}\sqrt{\frac{\pi {\varepsilon }_{p}{\varepsilon }_{0}{V}_{g}}{e{t}_{2}}}$$

where *v*_f_ = 10^6^ m/s is Fermi velocity, *ε*_p_ is relative permittivity of spacer, *ε*_0_ is vacuum permittivity, *V*_g_ is external voltage. So the graphene’s surface conductivity is descried by the Drude model at infrared region^42^:

5 $${\sigma }_{g}(\omega )=\frac{{e}^{2}{E}_{{\rm{f}}}}{\pi {{\rm{\hbar }}}^{2}}\cdot \frac{i}{\omega +i{\tau }^{-1}}$$

Here, $\tau =\mu {E}_{{\rm{f}}}/(e{v}_{{\rm{f}}}^{2})$ is the carrier relaxation time relating to the carrier mobility *μ* = 20000 cm^2^ V^−1^ s^−1^. Then the relative permittivity of graphene can be written as

6 $$\varepsilon (\omega )=1+i\frac{{\sigma }_{g}(\omega )}{{t}_{g}{\varepsilon }_{0}\omega }$$

where *t*_g_ = 0.5 nm is the thickness of graphene film. According to the above equations, the different values of the permittivity of the graphene can be obtained.

**Figure 2:**
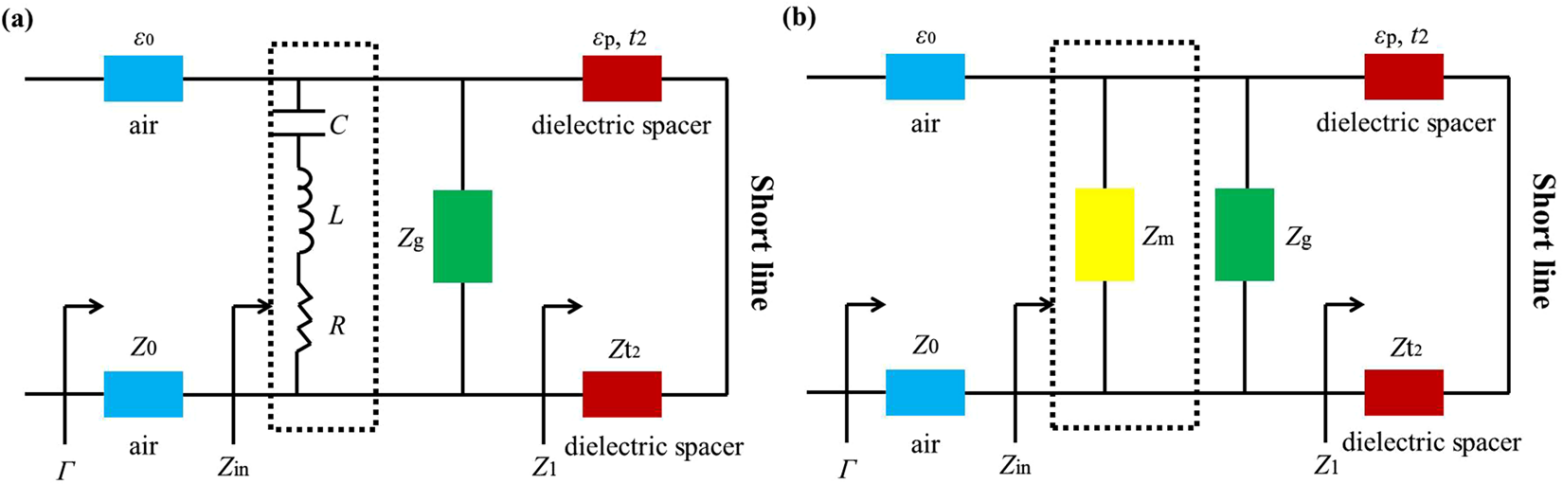
(**a**) Equivalent circuit model for the graphene-based MA. **(b**) Simplified model.

According to the transmission line theory, an equivalent circuit model (ECM) is presented in Fig. 2(a). In the circuit model, the bottom metal can be regarded as a short transmission line. *Z*_t2_ is the surface impedance of dielectric spacer. The top patterned metal array can be modelled by *R*-*L*-*C* circuit network. The resistance *R* and inductance *L* are induced by the metal, meanwhile capacitance *C* is caused by the gaps between the metal. But in the THz regime, the influence of *R* can be ignored. In Fig. 2(b), both the sheet impedances of the graphene and metamaterial can be regarded as *Z*_*g*_ and *Z*_*m*_, respectively. The input surface impedance of dielectric spacer *Z*_1_ can be shown as follows:

7 $${Z}_{1}=j{Z}_{t2}\,\tan ({k}_{m}{t}_{2})$$

where ${Z}_{t2}=\,\omega {\mu }_{0}/{k}_{m}$ and ${k}_{m}=\sqrt{{k}_{p}^{2}-{k}_{0}^{2}si{n}^{2}\theta }$ are the sheet impedance of dielectric spacer and the propagation constant along z axis. *k*_0_ and ${k}_{p}=\omega \sqrt{{\mu }_{0}{\varepsilon }_{p}}$ are the wave number of the incidence in free space and effective medium. *μ*_0_ is vacuum permeability. Based on the effective medium theory^43^, the imaginary part of impedance must be equal to zero at the resonant point. So the corresponding reflection efficiency *Γ* is expressed by

8 $$\Gamma =\frac{Re\{{Z}_{in}\}-{Z}_{0}}{Re\{{Z}_{in}\}+{Z}_{0}}$$

where *Z*_0_ is the air impedance (≈377Ω) and $\frac{1}{{Z}_{in}}=\frac{1}{{Z}_{g}}+\frac{1}{{Z}_{m}}+\frac{1}{{Z}_{1}}$ is the total input impedance of the MA, respectively. To investigate the absorption property, the expression of the sheet impedance of the graphene *Z*_g_ and metamaterial *Z*_m_ should be solved. Thus the effective impedance *Z*_m_ can be expressed as following^40^:

9 $${Z}_{m}\approx \frac{{Z}_{0}}{\frac{2\sqrt{{n}_{1}{n}_{2}}}{T}-({n}_{1}+{n}_{2}\,)}$$

where *n*_1_ and *n*_2_ are the refractive indices of the air space and graphene layer. The sheet impedance of the graphene Z_g_ can be expressed as:

10 $${Z}_{g}=\frac{1}{{\sigma }_{g}}$$

## Results and Discussion

**Figure 3:**
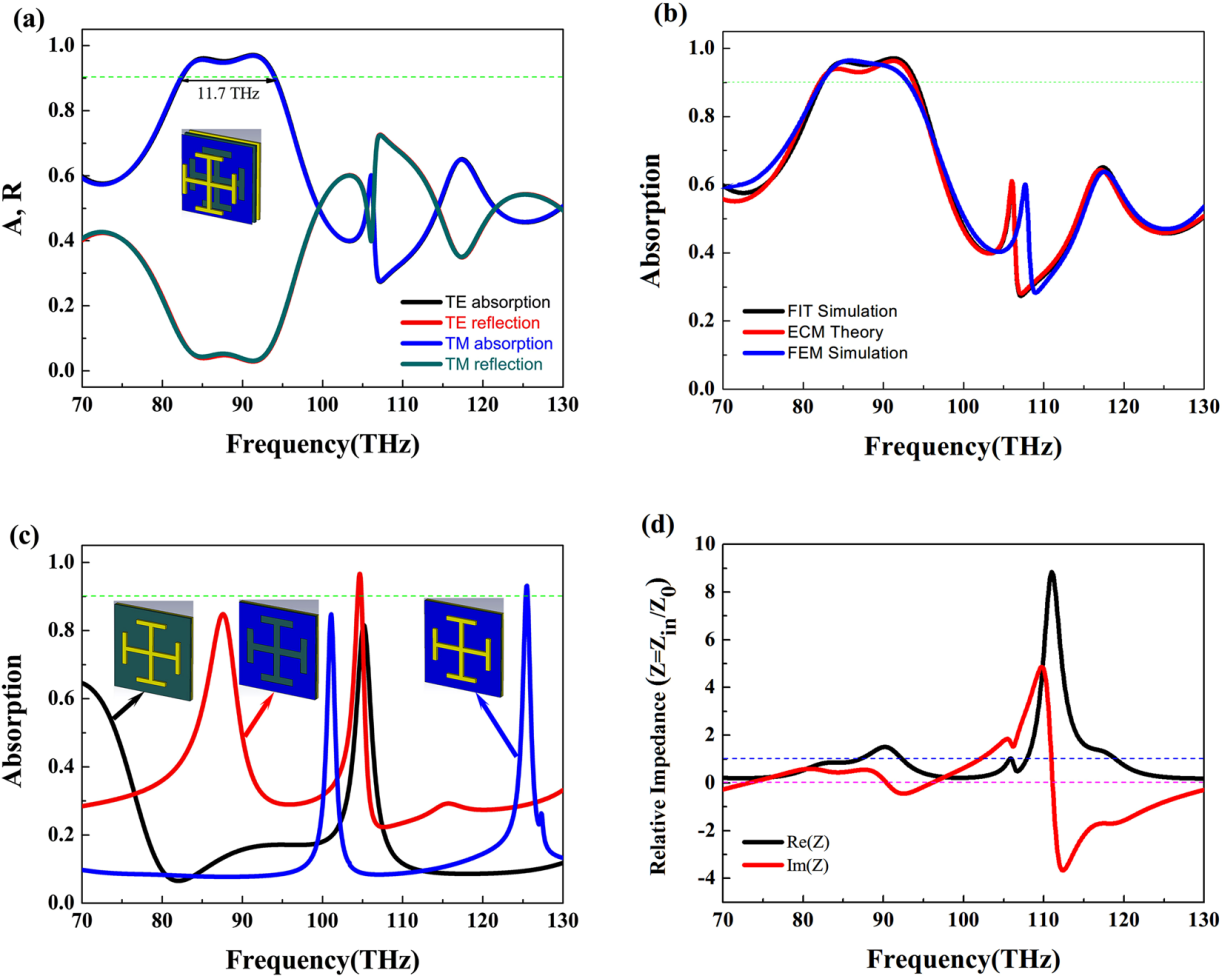
(**a**) Absorption spectra of the proposed MA. (**b**) Comparison between simulated and theoretical absorption coefficients. (**c**) Three comparison absorbers at normal incidence. (**d**) The total input impedance of the MA.

Figure 3(a) shows the absorption and reflection curves of the proposed MA under normal incidence with a fixed Fermi energy of 0.4 eV. The absorption over 90% can be achieved in the range of 82.3 to 94.0 THz. Also there is no obvious difference between transverse electric (TE) and transverse magnetic (TM) polarization wave due to the symmetric structure. In Fig. 3(b), we can see that the absorption curve of theoretical results are in good agreement with that of simulation data, and demonstrating the most-used ECM^44^ is accurate in the proposed MA. We see that there is also a good spectral overlap between the FIT and FEM simulation curves, which can further confirm the validity of our numerical results. The absorption curves of three comparison absorber are shown in black line (structure 1, JC metal + dielectric spacer + gold ground plate), red line (structure 2, slot graphene layer + dielectric spacer + gold ground plate) and blue line (structure 3, JC metal + continuous graphene layer + dielectric spacer + gold ground plate) in Fig. 3(c), respectively. It is found that the structure 1 shows only one resonance peak with the relatively low absorption efficiency of 81.6% at 105.16 THz, and a dual band absorber can be easily obtained by using the structure 2. The absorption bandwidth of the structure 3 is still narrow as compared with that of the proposed structure. So the high absorption is caused by the coupling effect between the slot graphene and JC metal. Then the total input impedance of the MA is discussed. The impedance of MA (*Z*_m_) and dielectric spacer (*Z*_1_) are considered as a whole and shown by *Z*_m1_. Under this assumption, the expression of total input impedance (*Z*_in_) can be changed as follows:

11 $$\frac{1}{{Z}_{in}}=\frac{1}{{Z}_{g}}+\frac{1}{{Z}_{m1}}$$

The relative impedance of the proposed MA (*Z* = *Z*_in_/*Z*_0_) is shown in Fig. 3(d). We can find that the real part is near unity (Re(*Z*) = 1), and the imaginary part comes to zero (Im(*Z*) = 0) at the resonant points *f*_1_ = 84.2 THz and *f*_2_ = 91.2 GHz. Therefore at these two resonance points, the total input impedance of the MA (*Z*_in_) is matched to the air impedance (*Z*_0_), and results in the high absorption in the absorber, while the real and imaginary parts fluctuate intensively in the frequency range of 105.9 to 130.0 THz, and yielding a relatively low absorption coefficient.

**Figure 4:**
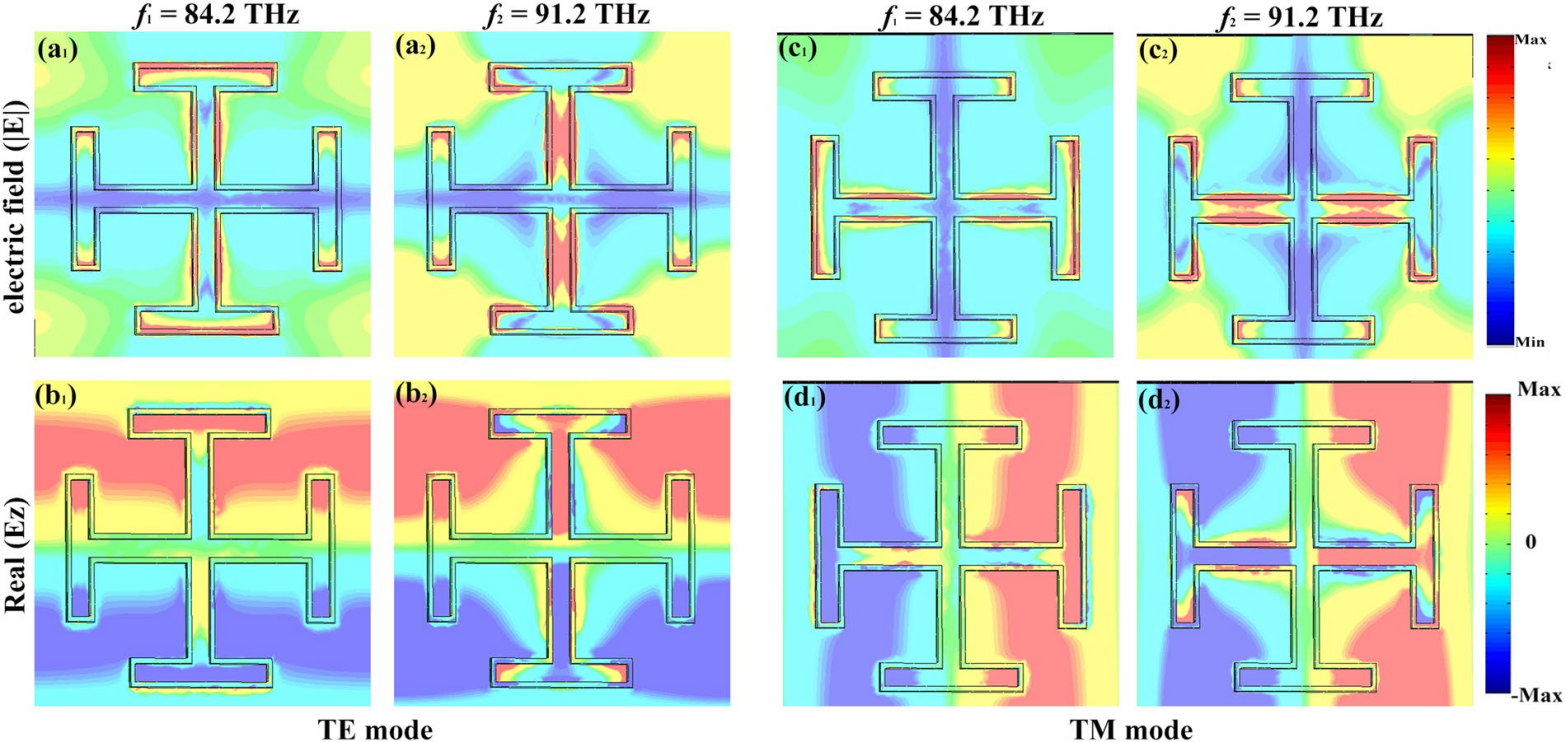
The distributions of the electric field (|E|) and real (Ez) at the resonance points.

In order to interpret the absorption mechanism of the MA, we investigate the electric field distributions for normal incidence at 84.2 THz and 91.2 THz. In Fig. 4(a_1_) and (a_2_), for TE polarized wave, it can be seen that the electric field mainly distributes on the edge of the JC metal and slot graphene layer, which means that the electric field is resonantly localized and concentrated at some part of the MA at low resonance point. And at high frequency, the electric field is resonantly localized and concentrated at some part of the metal and graphene. The corresponding z-component electric field distributions are shown in Fig. 4(b_1_) and (b_2_), which demonstrates that the opposite surface charges mainly distribute at the gap between the metal and slot graphene layer. With the frequency increases, the localized electric field moves to the surface of metal and some part of slot graphene layer, and the strength in the gap is stronger than that of the other areas. The analysis of the electric field distributions for TM is similar to that in TE mode (as shown in Fig. 4(c_1_,c_2_,d_1_,d_2_), which can be regarded as that in TE mode when the MA is rotated 90° around the *z*- direction.

**Figure 5:**
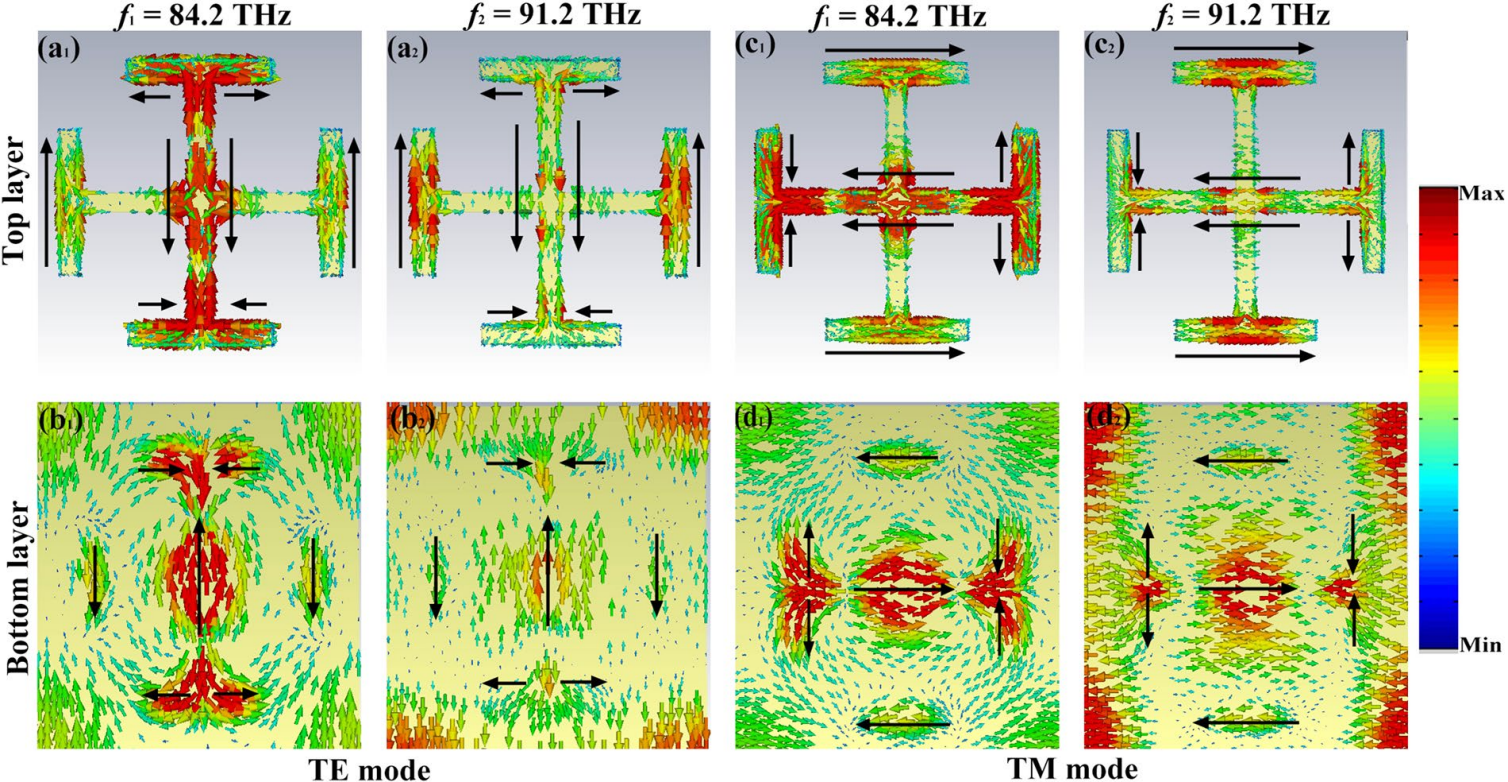
The distributions of surface currents on the top and bottom layer at the resonance points.

Then the surface current distributions of the MA at resonant peaks are shown in Fig. 5. Figure 5(a_1_,a_2_,b_1_,b_2_) show the surface current distributions on top and bottom layer under TE polarized wave, respectively. It is clearly observed that the surface currents on the top layer mainly flow along the direction of arrows, and on the bottom layer that is in the opposite direction. Therefore the anti-parallel surface currents form an equivalent current loop, which can exhibit magnetic dipole resonance under normal incidence. Meanwhile some surface currents in the top layer are parallel to that in bottom layer at some part of the absorber, and inducing the electric resonance. In Fig. 5(c_1_,c_2_,d_1_,d_2_), for the TM wave, the surface current distributions on the top and bottom layer are also similar to that in TE polarized wave. So the electric and magnetic resonances excite the high absorption at resonance points. This is another important factor in the whole absorption process.

**Figure 6:**
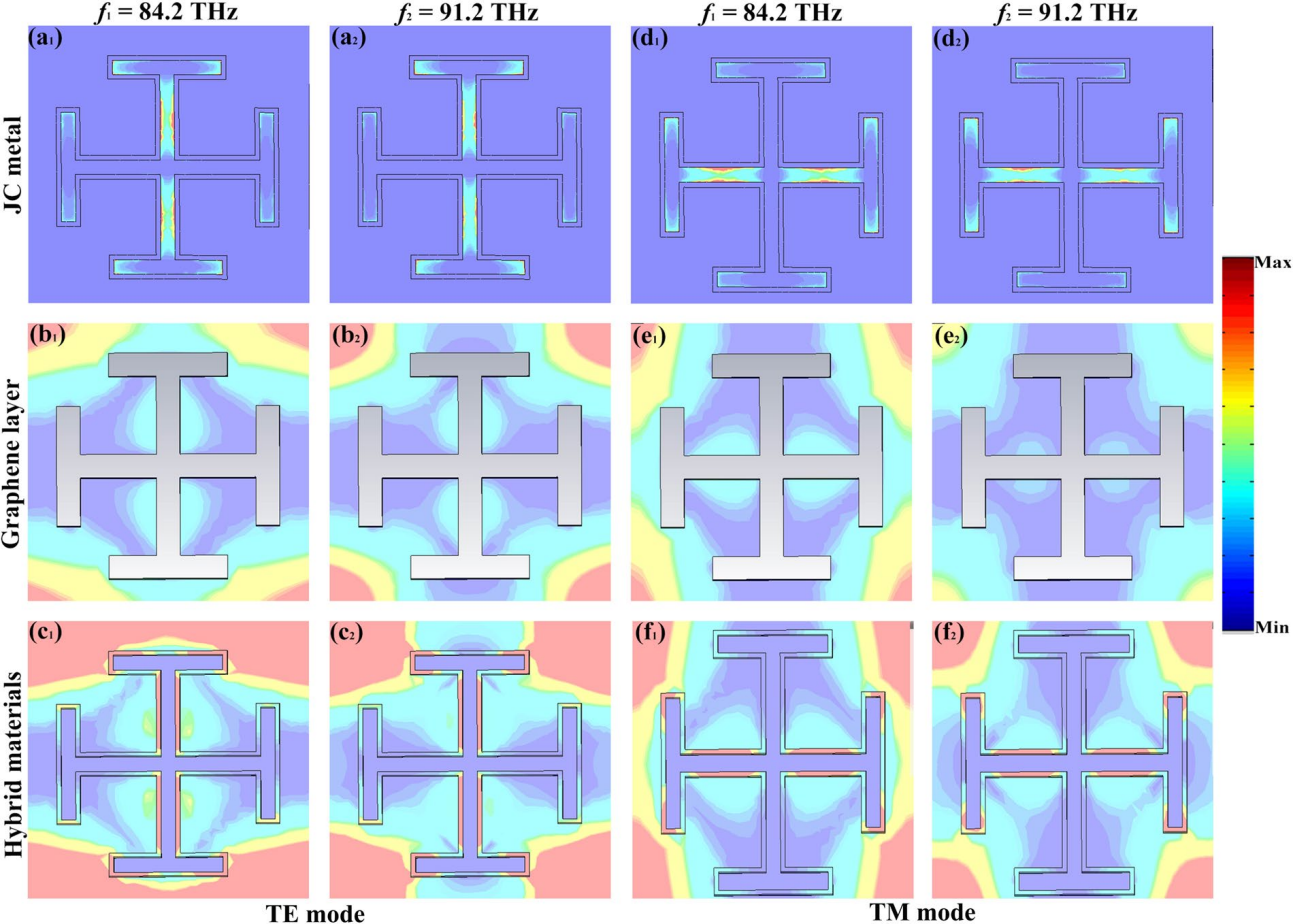
The power loss density distributions in the metal, graphene layer and proposed MA at the resonance points.

In order to gain more physical insight into the origin of the coupling effect between the metal and graphene, the power loss density distributions for metal and graphene at resonance points are shown in Fig. 6. Figure 6(a_1_) and (a_2_) show the power loss density distributions in the JC metal for TE polarized wave. We can see that the power loss density distributes on the edge of metal, which can be also found in the Fig. 4. Figure 6(b_1_,b_2_) show the power loss density distributions in the slot graphene layer under normal incidence. It is found that the distributions of power loss density mainly concentrate on the left and right sides of the slot graphene layer. The strength of the power loss destiny is further enhanced by using the hybrid material in the top layer as shown in Fig. 6(c_1_,c_2_). So the high absorption over 90% can be obtained in the proposed MA at the resonance points due to the coupling effect between the metal and graphene. For TM polarized wave, the power loss distributions are similar to that in TE polarized wave, as shown in Fig. 6(d_1_)–(f_2_).

**Figure 7:**
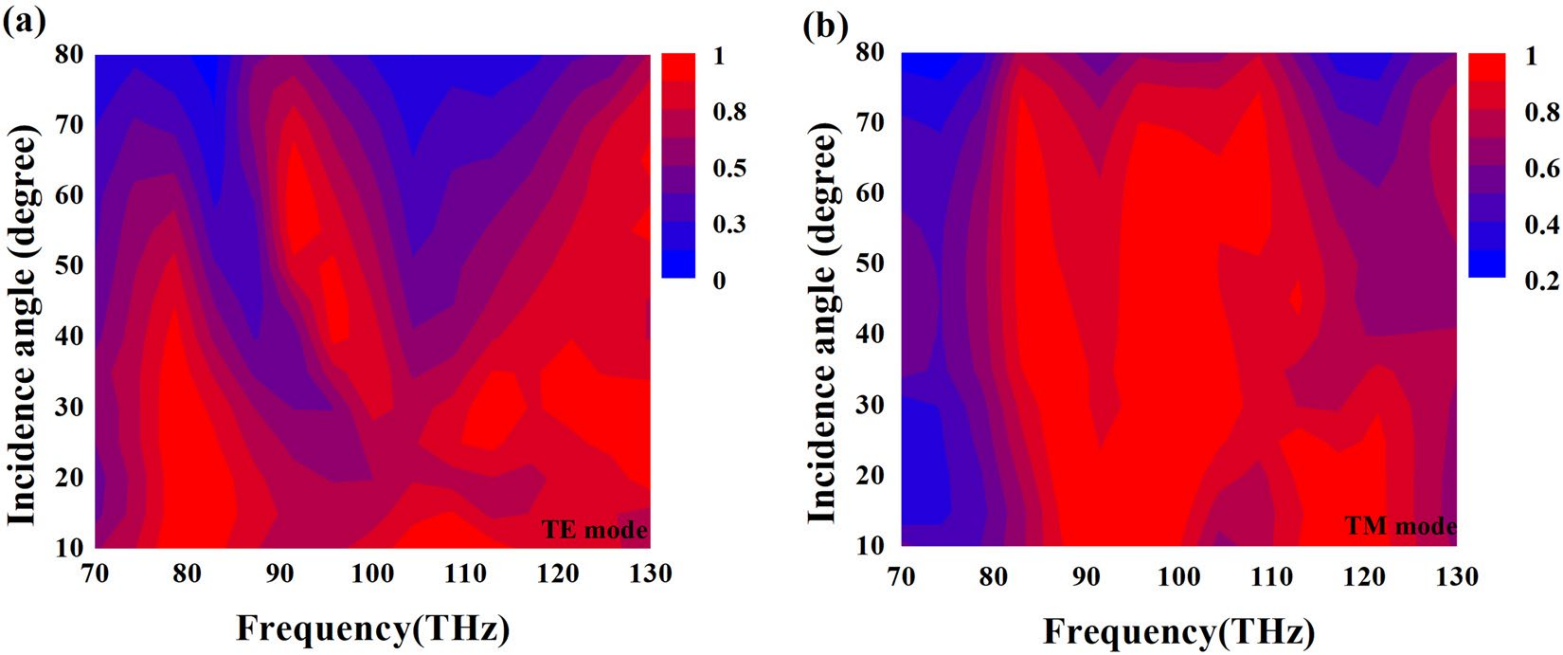
Simulation of angular dispersions of the absorption peaks for (**a**) TE wave and (**b**) TM wave.

The classical “two-mode waveguide coupling” model is used to explain the coupling effect. Based on the above discussion, the metal and graphene can be modeled as two waveguides, and satisfy the following coupled equations^45^:

12 $$\frac{d{E}_{a}}{dz}=-i{K}_{ab}{E}_{b}exp[-i({k}_{b}-{k}_{a})z]-i{C}_{a}{E}_{a}$$

13 $$\frac{d{E}_{b}}{dz}=-i{K}_{ba}{E}_{a}exp[-i({k}_{a}-{k}_{b})z]-i{C}_{b}{E}_{b}$$

where *E*_a_ and *E*_b_ represent the amplitudes of waveguide 1 (meal) and 2 (graphene). $${K}_{ab,ba}=\frac{\omega {\varepsilon }_{0}}{4}{\int }_{-\infty }^{\infty }{E}_{ay}{E}_{by}$$$$[{n}^{2}(x)-{n}_{a,b}^{2}(x)]dx$$ is the coupling coefficient describing the coupling strength between waveguide 1 and 2. $${C}_{a,b}=\frac{\omega {\varepsilon }_{0}}{4}{\int }_{-\infty }^{\infty }{({E}_{ay,by})}^{2}[{n}^{2}(x)-{n}_{a,b}^{2}(x)]dx$$ indicates the variation of propagation constants. To simplify the calculation, we set *C*_a_ = *C*_b_ and *K*_ab_ = *K*_ba_ = *K*. Based on this assumption, the power (*P*) of EM wave through the MA can be given by the following equations:

14 $${p}_{a}={|{E}_{a}(z)|}^{2}={|{E}_{a0}\frac{K}{{[{K}^{2}+{({\rm{\Delta }}k)}^{2}]}^{1/2}}\exp (-i{\rm{\Delta }}z)sin\{{[{K}^{2}+{({\rm{\Delta }}k)}^{2}]}^{1/2}z\}|}^{2}$$

15 $${p}_{b}={|{E}_{b}(z)|}^{2}={|{E}_{b0}\exp (-i{\rm{\Delta }}z)\{cos\{{[{K}^{2}+{({\rm{\Delta }}k)}^{2}]}^{\frac{1}{2}}z\}-i\frac{K}{{[{K}^{2}+{({\rm{\Delta }}k)}^{2}]}^{\frac{1}{2}}}\exp (-i{\rm{\Delta }}z)sin\{{[{K}^{2}+{({\rm{\Delta }}k)}^{2}]}^{\frac{1}{2}}z\}\}|}^{2}$$

where ${\rm{\Delta }}k=\frac{1}{2}[({K}_{a}+{C}_{a})-({K}_{b}+{C}_{b})]$ is the phase loss factor. In the case of ∆*k* ≫ *K*, the power can be exchanged between waveguide 1 and 2, it means that the interference effect between these two waveguides has taken place in the system, which is also seen in Fig. 3(c). The slot graphene layer can be regarded as metal due to its absorption curve (red line) is similar to that of metal (black line), and in the range of 82.3 to 94.0 THz, the absorption coefficient of slot graphene gets it maximum values, meanwhile that of JC metal can also obtain this in relative low values at the same range. Therefore the interference effect taking place within the structure can broaden the absorption band effectively. In Fig. 6, it can be found that the Joule heat inducing by the metal and graphene is beneficial to the broadband high absorption as well. Therefore we can draw a conclusion that the broadband high absorption is caused by the coupling between the interference effect taking place in the structure and Joule heat inducing by metal and graphene. Generally the high absorption should be maintained at a large incidence angle. Figure 7(a) and (b) present the absorption as a function of wavelength and angle of incidence for TE and TM wave, respectively. The incidence angle is varied from 0° to 80°. It is observed that the broadband absorption remains around 80% even at incidence angle of 80° for TE wave and 96% at 80° for TM wave over the entire infrared regime.

**Figure 8:**
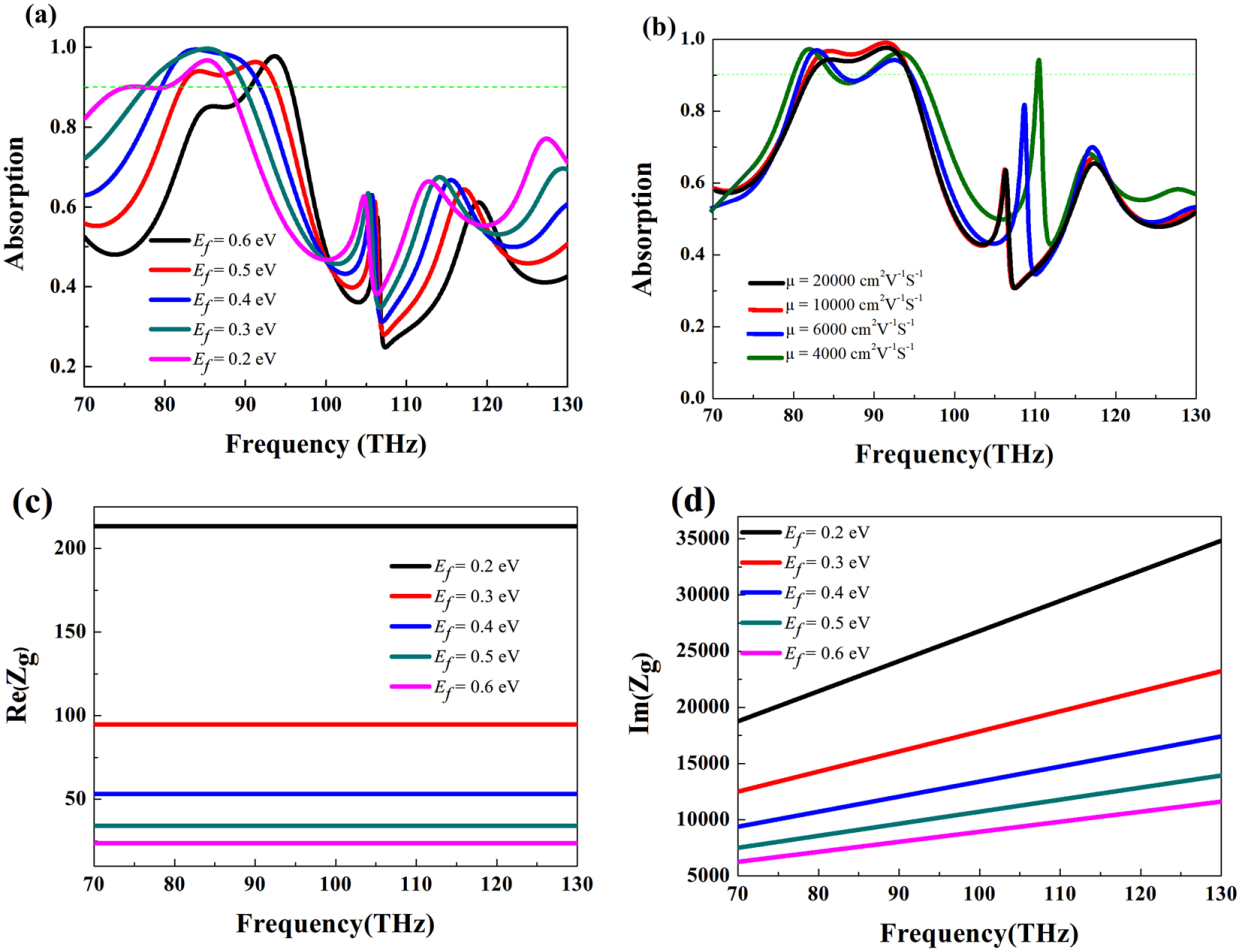
(**a**) The absorption curves of the proposed MA for (**a**) different Fermi energies under normal incidence and (**b**) different carrier mobility *μ*. Real part (**c**) and imaginary part (**d**) of *Z*_g_ versus frequency for different Fermi energies *E*_f_.

Figure 8(a) shows the absorption spectrum of MA with different Fermi energies under normal incidence. Graphene’s Fermi energies are set as 0.2 eV, 0.3 eV, 0.4 eV, 0.5 eV and 0.6 eV, respectively. It is seen that the position of the absorption peaks can be easily tuned by modifying the graphene’s Fermi energy, and with the Fermi energy increases, the resonant frequency is blue shifting. When the Fermi energy is 0.2 eV, the resonant frequency is located at 85.2 THz, and with the Fermi energy increases to be 0.6 eV, the corresponding resonant frequency is located at 93.6 THz. In addition, the high absorption over 90% will be maintained in the investigated frequency range. So the tunability of MA is realized via manipulating the Fermi energy of graphene layer through controlling external gate voltage instead of reconstructing or re-optimizing the geometry. Then the carrier mobility *μ* of the graphene layer is investigated because it is susceptible to the interface problem. Figure 8(b) shows the absorption spectrum with different carrier mobility. It is seen that with the carrier mobility decreases, the absorption notches become broader. The reason is that the lower mobility *μ* is, the lower intrinsic quality factor is^46^. Figure 8(c) and (d) show the calculated real and imaginary part of the surface impedance *Z*_g_ with different Fermi Level *E*_*f*_, respectively. It is found that the imaginary part of the *Z*_g_ continuously decreases with *E*_f_ increases and the real part of *Z*_g_ keeps constant when the frequency increases.

When graphene is placed in the vicinity of a metamaterial resonator, it will perturb the resonator. The change of the resonant angular frequency is given by^47^

16 $${\rm{\Delta }}\omega =({\rm{Im}}({\sigma }_{g}(\omega ))-i{\rm{Re}}({\sigma }_{g}(\omega )))\frac{{\int }^{}{|{E}_{xy}|}^{2}ds}{{W}_{0}}$$

where *σ*_g_(ω) is the conductivity of graphene, *S* is the area of graphene, *E*_xy_ is the electric field in the plane of graphene, *W*_0_ is the stored electromagnetic energy in an uncovered metamaterial resonator. Re and Im represent real and imaginary part of a complex value, respectively. From equation (16), we can find that the spectral shift of the resonance peaks Δ*ω* is determined by Im(σ_g_(ω)), whereas the amplitude modulation of the resonance peak is determined by the losses in graphene given by Re(σ_g_(ω)). At the infrared region, by manipulating graphene’s Fermi energy, it is possible to continuously adjust the absorption peaks and achieve the tunability of the MA.

**Figure 9:**
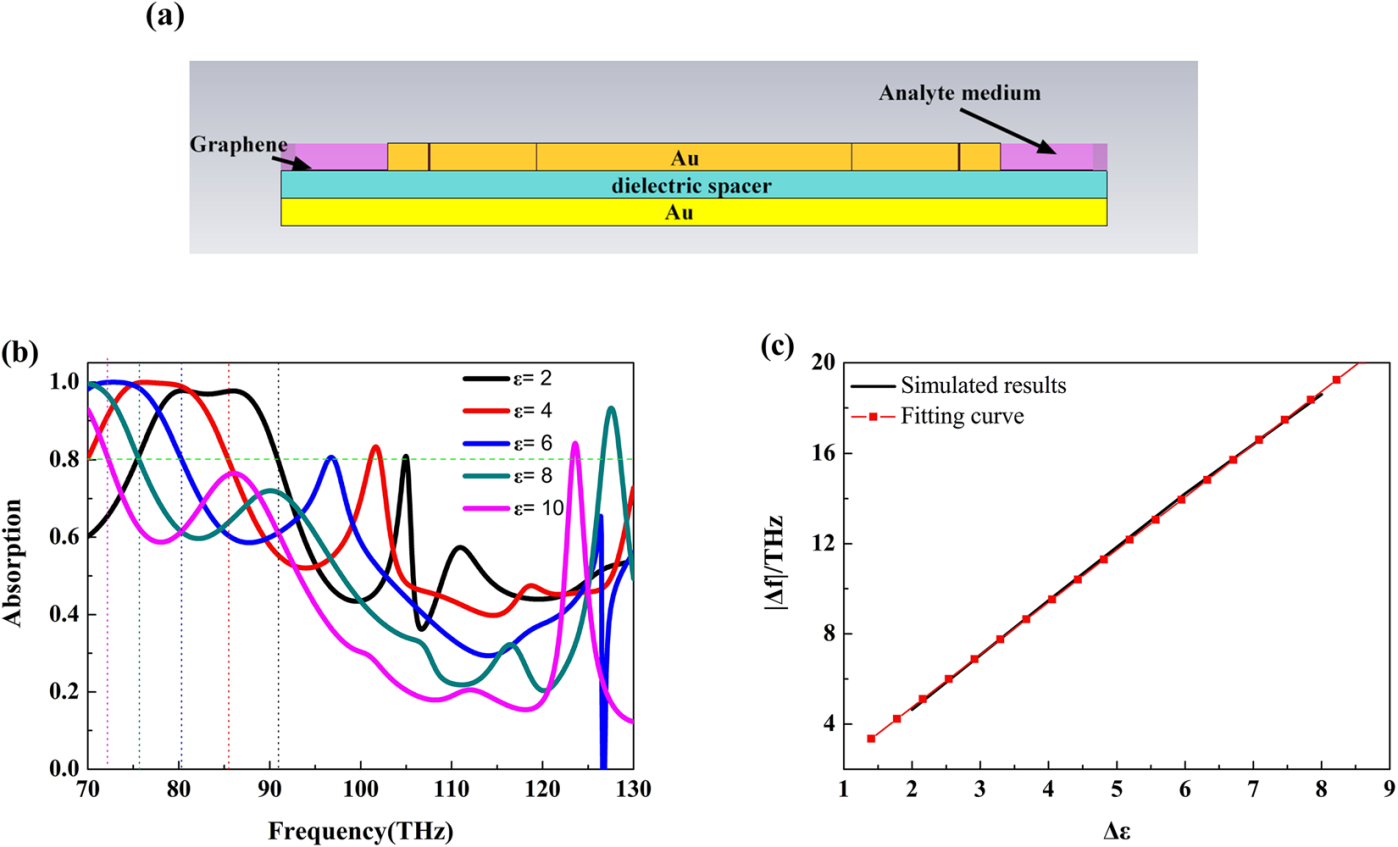
(**a**) The schematic of the structure for sensing application (purple part represents the analyte medium with the thickness of 0.1 μm). (**b**) The absorption curves of different permittivity analyte medium. (**c**) Simulated data and fitting curve.

Generally the sensitivity of permittivity sensors depends critically on the fact that the variation of permittivity of analyte medium can bring about the change in position of the 80% absorption point^48^. In Fig. 9(a), a 0.1-μm-thick analyte layer (purple part) with different permittivity (*ε*) varying from two to ten is placed onto the surface of graphene layer to fulfill this criterion. The corresponding absorption curves are shown in Fig. 9(b). We can see that the absorption bandwidth became narrow and the 80% absorption peak is red shifting as the permittivity values increases from two to ten. To investigate the sensitivity of the proposed MA, the relation curve between the frequency shift and different permittivity is shown in Fig. 9(c). By calculating the fitting curve, the peak frequency of 80% absorption increases linearly with the permittivity increases, indicating that the proposed MA can be used for permittivity sensing application.

We give a systematic comparison with the previous reported results in refs^48^ and^49^. The differences are summarized as follows: Firstly, the absorption efficiency of Fano-resonant metamaterials for the plasmonics-induced transparency (PIT) application is low, which limits the possibility of high absorption in MA, while the proposed structure is achieved and maintained this by using the tri-layer graphene-based MA. Secondly, the PIT is formed when the asymmetric structure is introduced into the system, while the proposed MA is a symmetric structure, which is simpler in experimental design. Thirdly, although the Fano-resonant graphene-based metamaterials have much higher qualify factor, the proposed system obtains the broadband high absorption and can be used to meet the requirement of perfect absorption in PIT devices.

## Conclusions

In summary, a tunable broadband graphene-based MA has been proposed numerically and theoretically at mid-infrared regions. Compared with the previously reported multiband graphene-based MAs, the proposed MA can obtain a broadband high absorption in the range of 82.3 to 94.0 THz via embedding JC metal into slot graphene layer, which is caused by the coupling effect between the metal and graphene, and this coupling effect is explained by two-mode waveguide coupling theory. The tunability of MA is obtained by manipulating the Fermi energy of the graphene layer through controlling external gate voltage instead of re-constructing the geometric structure. The ECM, distributions of electric field, surface current and power loss density are applied to explain the absorption mechanism. Further results show that the proposed MA can be used as a permittivity sensor. Therefore the proposed MA can find its potential applications in sensors and infrared absorbers.
